# Severe Postoperative Hemorrhage Following Dental Debridement in a Patient With PSPD

**DOI:** 10.1111/scd.70233

**Published:** 2026-08-08

**Authors:** Dan Burch, Richard Clark, Michael Bianchi

**Affiliations:** ^1^ St. Christopher's Hospital For Children Philadelphia Pennsylvania USA; ^2^ Drexel University College of Medicine Philadelphia Pennsylvania USA

**Keywords:** blood transfusion, general anesthesia, platelet storage pool deficiency, special care dentistry

## Abstract

**Background:**

Platelet Storage Pool Deficiency (PSPD) is a rare qualitative defect involving impaired granule secretion, often presenting with normal routine coagulation profiles. This case highlights the challenges of dental debridement in a young adult with special health care needs where functional platelet failure outweighs numerical counts.

**Case Description:**

An 18‐year‐old required dental debridement under general anesthesia. Despite a multidisciplinary preoperative protocol involving desmopressin and antifibrinolytics, the patient encountered refractory postoperative hemorrhage. The bleeding exceeded the capacity of local hemostatic measures, necessitating an emergent blood transfusion for stabilization.

**Practical Implications:**

This case underscores that pharmacological management of PSPD can be unpredictable. It reaffirms the necessity of the hospital‐based “Medical Home” for special care populations, ensuring immediate access to blood products and interdisciplinary expertise. Practitioners must look beyond standard lab values when planning invasive procedures for patients with qualitative platelet dysfunction.

## 1 Introduction

Platelet Storage Pool Deficiency (PSPD) represents a heterogeneous group of congenital qualitative platelet disorders characterized by a reduction in the number or functional capacity of intracellular secretory granules [1]. In patients with PSPD, the initial adhesion of platelets to the vascular subendothelium may occur, but the subsequent failure of the “release reaction” results in a fragile, ineffective clot that is prone to surgical disruption.

The clinical significance of PSPD in a dental setting lies in its unremarkable routine screening profile, which fails to capture qualitative primary hemostatic defects. While hemophilia involves deficiencies in plasma‐based clotting factors that are readily detected via prolonged activated partial thromboplastin time (aPTT), PSPD is a disorder of primary hemostasis [2]. This qualitative impairment is often not captured by standard screening assays, potentially leading to an underestimation of surgical bleeding risk [3]. Definitive diagnosis requires specialized functional or morphological assessments, as routine screenings may fail to detect the underlying defect [4].

For the patients requiring dental rehabilitation, these challenges are compounded by the necessity of general anesthesia (GA) and the highly vascular nature of the oral cavity. Managing an 18‐year‐old with PSPD requires an alternative approach to traditional factor replacement to a coordinated interdisciplinary, proactive hemostatic protocol. The fibrinolytic environment of the oral cavity, driven by salivary enzymes, further threatens the integrity of an already compromised platelet plug, making intensive periodontal procedures across highly inflamed vascular beds a high‐risk endeavor [5].

The objective of this case report is to illustrate the inherent unpredictability of bleeding risk in a patient with Platelet Storage Pool Deficiency (PSPD) undergoing dental debribement under GA. By tracing the clinical course from extensive preoperative planning to unanticipated intraoperative hemorrhage requiring transfusion, this report highlights the limitations of perioperative management strategies and clarifies the points at which rapid specialty consultation escalation becomes essential. In doing so, it reinforces the critical role of the hospital‐based “Medical Home” in managing medically fragile patients whose hemostatic stability cannot be reliably predicted or fully controlled.

## 2 Case History Report

An 18‐year‐old male with a confirmed diagnosis of Platelet Storage Pool Deficiency (PSPD) was referred for comprehensive dental treatment due to documented behavioral barriers that prevented routine outpatient care. The patient lived with his mother in a low socioeconomic environment but with ample access to medical services in the community. This patient was diagnosed with Hermansky‐Pudlak Syndrome (HPS), autism, and failure to thrive in 2013 at a different regional medical center.

Previous dental history included dental rehabilitation last completed within our hospital on August 12^th^, 2019 without any intra‐ or post‐operative complications. Three follow‐up visits were attempted within 30 days post‐procedure but failed due to behavioral issues. No dental services were received within our system from 2019–2026. The mother stated that examinations were attempted yearly in private practice dental offices but typically failed due to uncooperative behavior.

On January sixth, 2026, an initial dental examination and treatment planning was attempted but failed due to extreme anxiety, combative behavior, and refusal to participate in examination, resulting in a documented Frankl Behavior Rating of 1 (definitely negative). Completion of the dental examination and treatment planning were to be accomplished under general anesthesia due to patient and staff safety concerns.

The patient's medical history was notable for PSPD of the delta subtype, previously diagnosed after episodes of mucocutaneous bleeding. The diagnosis and subtype designation were established at an outside medical center based on prior hematology evaluation; however, the original raw data (e.g., platelet aggregation tracings and confirmatory ultrastructural findings) were not available for review in the current record. Accordingly, interpretation of perioperative bleeding risk in this report relies on the documented historical diagnosis and the observed clinical phenotype rather than repeat confirmatory testing. Although standard coagulation studies such as prothrombin time (PT), activated partial thromboplastin time (aPTT), and international normalized ratio (INR) had historically fallen within reference ranges, the patient's hematology team emphasized that PSPD represents a qualitative platelet defect with unreliable platelet plug formation and potentially unpredictable perioperative bleeding. On January 24^th^, 2026, baseline laboratory values were taken and made available. Results included a hemoglobin of 12.3 g/dL, hematocrit of 36.1%, platelet count of 197 × 10^3^/µL, a PT of 13.4 seconds, an INR of 0.9, and an aPTT of 34 seconds.

In anticipation of surgical care, extensive interdisciplinary coordination between dentistry, hematology, and anesthesiology teams was undertaken to develop a tailored perioperative management protocol aligned with evidence‐based recommendations for qualitative platelet disorders. On January 24^th^, 2026, hematology recommended treatment under GA with TXA availability, desmopressin (DDAVP) consideration, and blood products immediately accessible. Hematology's instructions were for the patient to take Amicar the night prior, morning of, then every 6 h for 5 days following procedure. Since behavioral barriers prevented meaningful in‐clinic assessment, only a limited extraoral visual inspection could be completed preoperatively, revealing a copious amount of supragingival calculus that covered the occlusal surface of several teeth. To preemptively counteract the highly fibrinolytic environment of the oral cavity and provide a stabilizing dosing rationale for the initially fragile platelet plug, the patient took 2 g of aminocaproic acid the night prior to and the morning of surgery, per the explicit timing and directions from his Hematologist.

In the operating room on January 28^th^, 2026, after induction of GA and securing a nasal endotracheal airway, a complete dental examination was performed. Intraorally, the patient exhibited copious plaque and calculus deposits and generalized severe gingivitis affecting marginal and papillary tissues. Gingival tissues were noticeably erythematous and edematous, with bleeding provoked by even light instrumentation. A full‐mouth radiographic series obtained under anesthesia confirmed the absence of dental caries, periapical pathology, or bony abnormalities. No restorative needs, extractions, or pulp therapy were indicated. Under anesthesia, full‐mouth scaling and debridement were performed. Hemostasis was achieved intraoperatively using direct pressure alone, and no topical hemostatic agents were required at that time. Estimated intraoperative blood loss was 50 mL, calculated via fluid‐volume subtraction of the ultrasonic scaler irrigant effluent collected within the surgical suction canisters. While an isolated 50 mL blood loss is hemodynamically negligible in a young adult, this minimal baseline bleeding underrepresented the true underlying hemorrhagic threat, serving as a false indicator of stability before secondary clot breakdown occurred. The patient was extubated uneventfully and transferred to the Post‐Anesthesia Care Unit (PACU) for close observation, in accordance with the recommended practice of extended monitoring for PSPD patients undergoing oral procedures.

**FIGURE 1:**
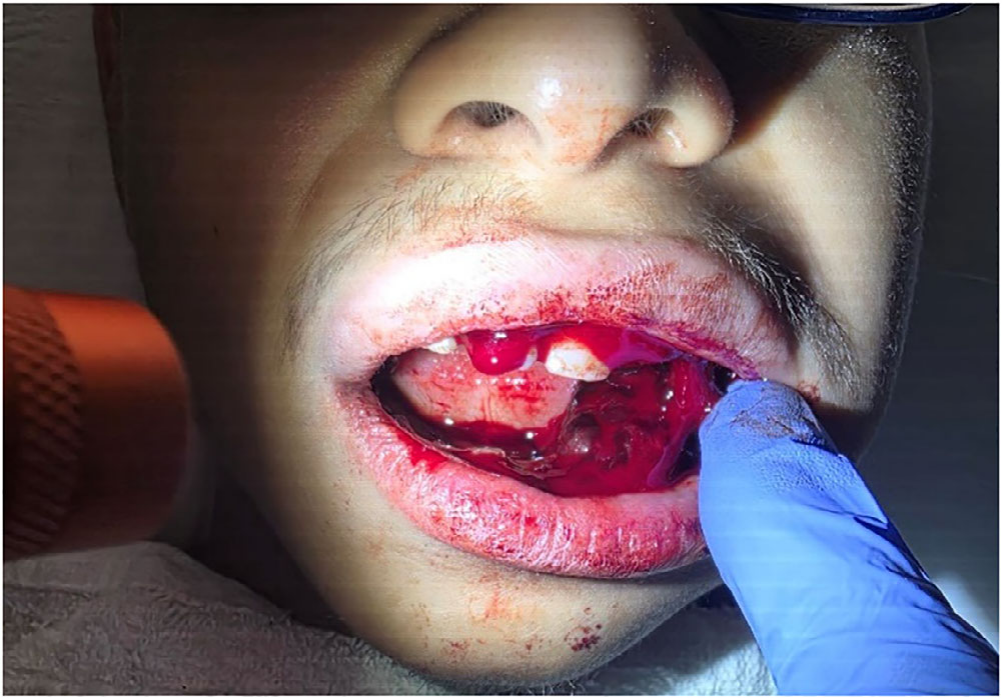
Refractory postoperative hemorrhage in the PACU. Intraoral view shortly after full‐mouth debridement demonstrating diffuse, profuse bleeding requiring escalation beyond local measures.

Shortly after arrival in the PACU, the patient developed significant postoperative oral hemorrhage that exceeded the degree observed intraoperatively (Figure 1). This acute hemorrhagic event explicitly demonstrated the limitations of standard pharmacological prophylaxis; despite adherence to the preoperative systemic antifibrinolytic regimen, the patient's severe qualitative defect precipitated a failure of hemostasis. When initial attempts at hemostasis using direct pressure were insufficient, the coordinated rescue protocol was rapidly escalated. He was given one unit of platelets (475 mL) along with DDAVP 0.3 mcg/kg administered via nebulizer—dosed to systematically maximize endothelial von Willebrand factor release. Shortly after, he was given an additional unit of platelets (225 mL). These efforts were unable to control his bleeding, and he was quickly transferred from the PACU to an in‐patient bed.

While on the in‐patient floor, a third unit of platelets (255 mL) was given along with TXA. TXA was administered at a dose of 500 mg/5 mL nebulized. Over time, bleeding diminished and ultimately resolved with the combined effects of TXA, transfusion, and sustained direct pressure with gauze. Despite these measures, the patient experienced significant blood loss, estimated at approximately 1300 cc. While diffuse capillary oozing across a vast surface area of inflamed gingiva rarely mirrors the macrovascular blood loss seen in major surgical procedures, the patient's qualitative platelet failure precipitated an acute hypovolemic challenge. This massive volume loss required the urgent transfusion of 460 mL of packed red blood cells (10 mL/kg) to restore circulating volume and oxygenation. Airway integrity remained stable, and the patient did not experience hypotension or oxygen desaturation. The patient remained under extended observation for 23 h, during which no recurrent bleeding episodes occurred.

**FIGURE 2:**
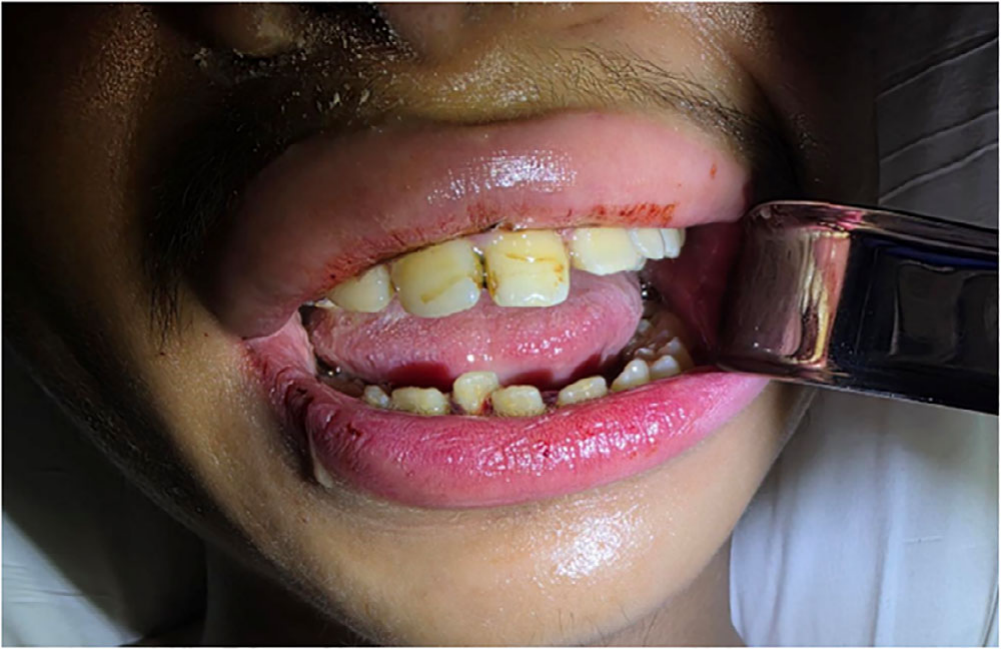
Postoperative clinical follow‐up (5 Days). Clinical presentation of the maxillary and mandibular arches five days after the hemorrhagic event. The gingival tissues appear fully healed with a significant reduction in the generalized inflammation and edema observed preoperatively. There is no evidence of delayed bleeding or hematoma formation, and the patient reported no further discomfort.

Upon stabilization, the patient was discharged home with instructions for a soft diet for seven days, avoidance of vigorous oral activity, and a prescription for 50 mg/kg aminocaproic acid taken by mouth every 6 h for 5 days to sustain systemic fibrinolysis inhibition. At the postoperative follow‐up appointment (five days post‐surgery), the gingival tissues appeared fully healed, with no evidence of ongoing inflammation, infection, or delayed bleeding as seen in Figure 2. After stabilization from this significant hemorrhagic complication, the patient was placed on a cautiously structured, hospital‐coordinated periodontal maintenance program aimed at minimizing future bleeding risk while maintaining periodontal health. Maintenance visits were scheduled at shortened recall intervals of 6 weeks, with ongoing reassessment based on clinical stability and hemostatic response. To overcome the documented behavioral barriers without resorting to repeat general anesthesia, these visits utilized structured behavioral desensitization, positive reinforcement, familial modeling, and diazepam 10 mg as an anxiolytic. Preventive care centered on careful supragingival plaque control, reinforcement of atraumatic oral hygiene practices, and strict limitation of subgingival instrumentation to situations of clear clinical necessity. Risk mitigation strategies prioritized early identification of gingival inflammation, avoidance of procedural trauma, and continuous communication with the hematology team. This surveillance‑based approach reflects the necessity of emphasizing disease prevention and conservative management in patients with PSPD following a major bleeding event.

## 3 Discussion

### 3.1 Pathophysiology of Hemostatic Failure in Delta‐Granule Deficiency

The profound hemostatic failure observed postoperatively is fundamentally rooted in the pathophysiology of delta‐granule deficiency. Delta granules, or dense bodies, serve as the primary reservoir for essential small‐molecule agonists, including adenosine diphosphate (ADP), ionized calcium, and serotonin [1]. Upon initial vascular injury and subsequent platelet activation, the exocytosis of these constituents is mandatory for the autocatalytic amplification of the hemostatic response [2]. These agonists mediate the secondary recruitment of circulating platelets to the site of injury and are vital for the “second wave” of aggregation. In PSPD, the absence or reduction of these granules attenuates this recruitment phase, resulting in a primary hemostatic plug that is structurally fragile and poorly contracted [1, 4]. This thrombus instability is particularly precarious in the oral cavity, where high fibrinolytic activity and mechanical tongue movement can easily dislodge a poorly consolidated clot, leading to the delayed and refractory hemorrhage characterized in this case [5].

### 3.2 Recent Advances in Platelet Function Tests

Recent diagnostic trends emphasize whole‐blood and point‐of‐care assays as adjuncts to traditional platelet testing. Multiple electrode aggregometry (MEA; Multiplate) can provide rapid perioperative insight using whole blood and small sample volumes but its sensitivity for mild qualitative platelet disorders is limited and may fail to capture features classically identified on light transmission aggregometry (LTA) [6, 7, 8, 9].As a result, MEA may suggest global hemostatic impairment but does not replace gold‐standard diagnostic approaches such as LTA and electron microscopy for granule disorders [2, 4]. Similarly, VerifyNow is optimized for monitoring antiplatelet therapy rather than diagnosing hereditary platelet pathologies [10, 11]. Accordingly, in suspected storage pool defects, point‐of‐care assays should be interpreted as supportive rather than definitive, with diagnostic confirmation still relying on specialized functional and/or morphological testing [2, 4, 8].

### 3.3 Rationale for Omission of Manual Bleeding Time Testing

The omission of historical manual bleeding time tests, such as the Ivy or Duke methods, warrants specific clinical mention. While standard plasma‐based coagulation profiles are unsuited for identifying qualitative primary hemostatic defects, manual skin‐incision bleeding tests have been phased out of modern academic hospital centers due to low sensitivity, high operator variability, and potential for permanent scarring. Furthermore, attempting a manual cutaneous bleeding time test preoperatively was entirely precluded by the patient's extreme dental anxiety, uncooperative behavior, and documented baseline behavioral barriers (Frankl Behavior Rating 1).

### 3.4 Developmental and Behavioral Considerations for GA

The patient's age (18 years) and inability to tolerate outpatient care necessitated a hospital‐based (GA) approach. Documented behavioral barriers prevented routine hygiene and contributed to severe gingival inflammation, which amplified bleeding risk; once scaling began, the patient's qualitative platelet defect could not compensate for the vascular trauma in inflamed tissues, leading to the refractory hemorrhage observed. At the onset of adolescence, the psychological burden of extensive oral surgery can be profound. Utilizing GA in a hospital setting not only addresses dental anxiety but, more importantly, provides a controlled environment for hemodynamic monitoring and airway protection—a necessity when managing potential bleeding diatheses in the oropharyngeal space [12].

### 3.5 Gingival Inflammation as a Bleeding Risk Factor

In the context of qualitative platelet disorders such as PSPD, localized periodontal inflammation acts as a profound amplifier of perioperative bleeding risk [2]. The resulting marginal and papillary tissues were highly edematous, erythematous, and extremely friable upon minimal instrumentation. While an intact hemostatic system can typically resolve the minor microvascular trauma associated with routine dental debridement, patients with PSPD are severely disadvantaged by the absence of alpha or delta granules, which are necessary for the secondary wave of platelet aggregation and stabilization of the fibrin clot [1, 2].

The marked hypervascularity and vasodilation characteristic of chronic gingival inflammation dramatically increase the local hemostatic burden. In this patient, the generalized inflammatory state functioned as the primary catalyst for the subsequent hemorrhagic crisis. The qualitative platelet defect could not compensate for the extensive vascular trauma induced by scaling highly engorged tissues, ultimately leading to the refractory postoperative bleeding observed. This fragile hemostatic balance is further compromised by the oral cavity's inherently fibrinolytic environment, where salivary enzymes actively threaten the integrity of an already weak platelet plug [5]. Consequently, clinicians must recognize that in populations with qualitative platelet dysfunction, untreated gingival inflammation effectively transforms conservative non‐surgical interventions, such as scaling, into high‐risk surgical events.

### 3.6 Limitations of Pharmacological Prophylaxis in PSPD

Current literature often highlights the efficacy of DDAVP and antifibrinolytics in managing mild‐to‐moderate qualitative defects [3, 13]. However, this case illustrates a critical failure of the standard tri‐partite protocol (DDAVP, TXA, and local hemostatics). Despite the preoperative administration of DDAVP to enhance von Willebrand factor levels, the patient still experienced a profound secondary hemorrhage [13]. This clinical course highlights that rather than waiting for postoperative hemorrhage to manifest, clinicians should proactively integrate local chemical adjuncts—such as immediate post‐scaling rinses or prolonged pressure compression with 4.8% topical tranexamic TXA gauze—directly into the immediate post‐operative workflow within the operating room. This directly protects the tenuous platelet‐fibrin interface across the vast, irritated capillary bed before secondary clot degradation initiates. This suggests that in some PSPD subtypes—particularly those with severe delta‐granule depletion—the “stickiness” provided by DDAVP cannot compensate for the total lack of secondary signaling molecules (ADP, serotonin) required for a stable clot [1, 2, 14]. Clinicians must therefore view pharmacological agents as a means to *reduce* risk, rather than a guarantee of hemostasis, and must be prepared for emergent transfusion if local and systemic measures are exhausted [2, 15]. Unlike Hemophilia, where factor levels are the primary concern, PSPD requires a focus on the quality of the initial platelet plug [3]. Therefore, the synergy between systemic DDAVP and aggressive local hemostatic measures (as outlined in Table 1) is the cornerstone of preventing secondary hemorrhage [5, 12].

**TABLE 1:** Multi‐phase clinical management protocol for dental rehabilitation under general anesthesia in patients with Platelet Storage Pool Deficiency (PSPD).

Phase	Category	Action items & considerations
Pre‐operative	Hematology consult	Confirm specific subtype of PSPD (Alpha, Delta, or Gray Platelet Syndrome). Review historical bleeding phenotype and previous response to hemostatic agents.
	Pharmacological trial	Perform a DDAVP challenge to assess platelet response. Establish baseline CBC, PT/PTT, and Platelet Function Analysis (PFA‐100).
	Logistics	Coordinate “Standby” availability of HLA‐matched platelet pheresis with the blood bank. Verify general anesthesia clearance and NPO status.
Intra‐operative	Systemic hemostasis	Administer DDAVP (0.3 mcg/kg) or antifibrinolytics (Tranexamic Acid/ aminocaproic acid) as per hematology protocol. Maintain meticulous blood pressure control to prevent “rebound” bleeding.
	Procedural technique	Use atraumatic extraction techniques (e.g., periotomes) and minimally invasive procedures (e.g., supragingival scaling). Prioritize primary closure of all surgical sites with resorbable sutures.
	Local measures	Apply topical hemostatic agents: Gelfoam, Surgicel, or Topical Thrombin. Use 4.8% TXA soaked gauze for immediate post‐extraction pressure.
Post‐operative	Immediate recovery	Monitor for hematoma formation or airway obstruction in PACU. Ensure 2–4 h of supervised observation before discharge.
	Home care	Prescribe liquid/soft diet for 7 days to avoid mechanical trauma to clots. Continue systemic antifibrinolytics for 5–7 days post‐surgery.
	Emergency plan	Provide 24/7 contact info for the Hospital Dental Resident and Hematologist on‐call.

*Note*: This table delineates a comprehensive, multidisciplinary framework for the perioperative management of patients with qualitative platelet defects. Given that standard coagulation profiles (PT/PTT/INR) often yield normal results in this population, the protocol emphasizes functional assessment and preoperative coordination between hematology, anesthesiology, and dental surgery. The phases outlined—ranging from the preoperative DDAVP challenge to the transition from pharmacological prophylaxis to transfusion rescue—serve as a clinical guide for mitigating the risk of refractory secondary hemorrhage in high‐risk special care patients.

### 3.7 Local Hemostatic Precision

#### 3.7.1 Clinical Framework for Invasive Procedures

In the surgical management of patients with inherited qualitative platelet disorders (QPDs), the literature emphasizes a hierarchical approach to local hemostasis, particularly during high‐risk interventions such as extractions. The standard of care traditionally involves the use of mechanical adjuncts, including resorbable gelatin sponges or oxidized regenerated cellulose, to provide a scaffold for the deficient platelet plug [2, 13, 15]. Furthermore, the literature highlights the utility of primary closure using non‐resorbable or slowly resorbable monofilament sutures to provide mechanical tamponade within extraction sockets, thereby shielding the fragile clot from the high fibrinolytic activity of saliva [3, 5, 16]. In such conceptual frameworks, chemical adjuncts—including topical thrombin or fibrin glues—are often utilized to compensate for the delayed “second wave” of aggregation characteristic of secretion defects [1, 17].

#### 3.7.2 Case‐Specific Application: Periodontal Scaling and Microvascular Integrity

While the broader literature addresses surgical extractions, the reported case presented a unique challenge involving diffuse microvascular trauma rather than discrete surgical sites. In this instance, the patient's dental rehabilitation focused exclusively on intensive periodontal scaling and root planing in the presence of severe gingival inflammation. Unlike the localized nature of a suture‐stabilized extraction site, scaling induces generalized capillary oozing across a vast surface area of inflamed marginal and papillary tissues.

Given the patient's PSPD, the absence of alpha and delta granules precluded the stabilization of the initial hemostatic plug across these diffuse scaling sites [1]. Consequently, management transitioned from the generalized background measures of systemic prophylaxis to high‐precision local intervention. This involved the application of prolonged mechanical pressure followed by the localized delivery of topical antifibrinolytics. Specifically, gauze soaks saturated with 4.8% tranexamic acid were applied to the quadrant‐specific scaling sites to inhibit local plasminogen activation and protect the tenuous platelet‐fibrin interface from premature degradation [18]. This case underscores that in the absence of extractions or suturable wounds, hemostatic precision in QPD management must focus on the rigorous control of the gingival inflammatory bed and the targeted use of topical agents to manage scaling‐induced hemorrhagic risk.

### 3.8 The Dental‐Medical Home Model: Systematic Risk Management in Complex Hemostasis

The Dental‐Medical Home (DMH) is defined as a coordinated, comprehensive, and continuous relationship between dental providers and the patient's medical subspecialties, typically integrated within a hospital‐based infrastructure [19, 20]. For patients with qualitative PSPD and significant behavioral barriers, this model serves as a functional necessity for mitigating high‐risk surgical outcomes.

In the reported case, the DMH framework facilitated rigorous preoperative planning through synchronous interdisciplinary coordination. This infrastructure allowed for the precise timing of pharmacological prophylaxis—specifically desmopressin and antifibrinolytics—alongside the preemptive preparation of type‐specific blood products, which is critical given the unpredictable nature of PSPD‐related bleeding [2].

The utility of this integrated care coordination was further validated during the patient's refractory postoperative hemorrhage in the post‐anesthesia care unit. The immediate proximity of specialized monitoring and an integrated laboratory network enabled a rapid escalation from local hemostatic measures to emergent blood transfusion. Such high‐acuity interventions are often unattainable in traditional community settings, where delays in laboratory assessment or patient transfer could result in significant morbidity. Consequently, the hospital‐based DMH model bridges the gap between dental rehabilitation and life‐saving medical intervention, ensuring a protective environment for medically fragile populations [21].

### 3.9 Limitations

A significant limitation of this case report is the reliance on historical diagnostic data to confirm the patient's diagnosis of PSPD. Because the initial diagnosis and subtype classification were established at a previous medical center, the primary raw data—specifically the original light transmission aggregometry (LTA) curves and confirmatory electron microscopy—were not available for primary review in the current hospital‐based record.

The identification of the delta‐granule subtype was based on historical hematology evaluations and a clinical history of recurrent mucocutaneous bleeding, which informed the multidisciplinary perioperative strategy. While the patient's profound refractory hemorrhage aligns with the secondary aggregation failure characteristic of secretion defects, the absence of electron microscopy to visualize the specific reduction in intracellular secretory granules prevents a definitive morphological quantification.

Despite these diagnostic constraints, the patient's clinical presentation serves as a critical example of the “diagnostic blind spot” inherent in qualitative platelet disorders, where profound functional failure can occur despite normal routine coagulation screening results (as described in the Case History Report). This case highlights the necessity of maintaining clinical vigilance and hospital‐based “Medical Home” access for patients with historical diagnoses of qualitative dysfunction, even when detailed original diagnostic criteria are inaccessible.

## 4 Conclusion

The management of this case illustrates that safe surgical care for patients with PSPD requires a carefully individualized hematologic strategy, while acknowledging the disorder's inherent unpredictability. Unlike quantitative coagulopathies, PSPD may not respond reliably to standard pharmacologic measures, and significant bleeding can occur despite thorough preparation, as demonstrated by the need for transfusion in this patient. The Hospital Dentist plays a critical role as the coordinator of multidisciplinary care, ensuring seamless collaboration and rapid escalation when complications arise. This experience reinforces the necessity of hospital‐based treatment for medically complex patients and underscores the importance of sustained clinical vigilance when laboratory findings do not accurately reflect bleeding risk.

## Funding

No external funding or commercial support was received for the preparation of this case report.

## Conflicts of Interest

The authors declare that there are no conflicts of interest, financial or otherwise, related to the material presented in this manuscript.
